# Draft genome sequences of *Salmonella enterica* subsp. *enterica* isolates from fresh produce and agricultural environments in South Korea

**DOI:** 10.1186/s13104-025-07494-8

**Published:** 2025-10-13

**Authors:** Su-Hyeon Kim, Ji Min Han, Gyu-Sung Cho, Erik Brinks, Charles M. A. P. Franz, Mi-Kyung Park

**Affiliations:** 1 School of Food Science and Biotechnology, and Food and Bio-industry Research Institute, Kyungpook National University, Daegu, 41566 Korea; 2 Department of Infectious Disease Healthcare, Kyungpook National University, Daegu, 41566 Korea; 3 Department of Microbiology and Biotechnology, Federal Research Institute of Nutrition and Food, Max Rubner-Institut, Hermann-Weigmann-Straße1, 24103 Kiel, Germany

**Keywords:** *Salmonella*, Antimicrobial resistance, Fresh produce and agricultural environment, South korea

## Abstract

**Objectives:**

*Salmonella enterica* is a globally significant foodborne pathogen and a leading cause of gastrointestinal infections, with increasing concern over strains harboring antimicrobial resistance (AMR). This Data Note reports draft genome sequences of six *S. enterica* subsp. *enterica* isolates from fresh produce and agricultural environments in South Korea. The objective of this work was to provide genomic data on environmental *Salmonella* isolates, their serovar diversity, AMR gene profiling, and genetic attributes relevant to *Salmonella* surveillance and comparative genomics.

**Data description:**

Draft genomes of six isolates consisted of 3 to 7 contigs with genome sizes ranging from 4.89 to 5.02 Mbp and GC content (%) between 51.89% and 52.24%. The isolates were identified as four serovars, including three *S*. Typhimurium, *S*. I 4,[5],12:i:-, *S*. Kentucky, and *S*. Montevideo, and five MLST types. Among them, three strains representing serovars including Typhimurium,I 4,[5],12:i:-, and Kentucky harbored acquired AMR genes, conferring resistance to aminoglycosides, aminopenicillins, diaminopyrimidines, sulfonamide, and tetracyclines. Our study highlights the genomic diversity and resistance potential of *S. enterica* isolates derived from preharvest agricultural environments, providing valuable resources for future comparative analyses and AMR surveillance efforts.

## Objective

*Salmonella enterica* is one of the most significant foodborne pathogens globally, responsible for hundreds of millions of infections and mortality each year [1]. Among various transmission routes, fresh produce and its surrounding environments have recently emerged as a significant source of *Salmonella* contamination. In the USA, fresh produce-associated *Salmonella* outbreaks accounted for more than 33.7% of all foodborne *Salmonella* outbreaks reported between 1998 and 2021 [2]. Among the more than 2610 known *Salmonella* (*S*.) *enterica* serovars, several serovars such as *S.* Typhimurium, *S.* Enteritidis, *S.* Bareilly, *S.* Weltevreden, *S*. Thompson, *S*. Newport, and *S*. Saintpaul have frequently been implicated in fresh produce-related outbreaks [2, 3]. These serovars demonstrate persistence in soil, irrigation water, and organic fertilizers, contributing to their relevance in preharvest contamination [1, 4]. Moreover, recent reports showed an increasing occurrence of AMR among such isolates [2].

In a previous study, our group isolated *Salmonella enterica* strains from fresh produce and agricultural environment samples in South Korea and assessed their AMR profiles [5]. While our previous study [5] focused on 16 S rRNA-based identification and phenotypic antibiotic resistance, the present study reports draft genome sequences of the isolates, accompanied by in silico predictions of serovars, sequence types (STs), and antimicrobial resistance gene profiles. The generated data are intended to support comparative genomic analyses and surveillance efforts related to *Salmonella* contamination in fresh produce and agricultural environments. The detailed isolation, biochemical identification, and sequencing procedures used for these isolates are provided in the Data Description section.

## Data description

A total of six *Salmonella* strains were isolated from green onion, peach leaves, peach orchard soil, and cow manure collected in Daegu and Gyeonsangbuk-do provinces, South Korea. Each sample (25 g) was pre-enriched in 225 mL of tryptic soy broth (BD Difco, Franklin Lakes, NJ, USA) at 37 °C for 18 h, following a previously validated laboratory protocol [5] based on Ministry of Food and Drug Safety (MFDS) guidelines [6] with minor modifications [7]. Then, 1 mL of the pre-enriched culture was transferred into Rappaport-Vassiliadis broth (BD Difco) at 42 °C for 24 h. Aliquots were streaked onto MacConkey and xylose lysine deoxycholate agar (BD Difco), and the plates were incubated at 37 °C for 48 h. Two colonies with characteristic morphology were selected and presumptively identified using indole, methyl red, Voges-Proskauer, and citrate tests, as well as 16 S rRNA gene sequencing [5].

Genomic DNAs were extracted using the Wizard^®^ HMW DNA Extraction Kit (Promega Co., Medison, WI, USA) according to the manufacturer’s instructions. DNA libraries were prepared using the ligation sequencing and native barcoding kit (SQK-NBD114.96, Oxford Nanopore Technologies Inc., Oxford, UK) and sequenced using ONT PromethION 2 Solo platform with R10.4.1 flow cell. Basecalling and demultiplexing were conducted using Dorado version 0.9.5. The raw sequence data (Data set 1) [8–13] were filtered using Chopper v. 0.9.2 with a minimum average quality score of Q15 and minimum length of 600 bp [14]. *De novo* assembly was then performed using Flye (v. 2.9.5) with the --nano-corr option [15], resulting in a coverage range of 66× to 198× (Data file 1) [16]. The quality of assembled contigs was assessed using QUAST v.5.3.0 [17]. Genomes were annotated using the NCBI Prokaryotic Genome Annotation Pipeline [18] and serotypes were predicted with SeqSero v 1.3.1 [19]. Average nucleotide identity (ANI) and dDDH were calculated using the pyani pipeline [20] and the genome-to-genome distance calculator (GGDC, formula 2) [21], respectively. Identification of acquired AMR genes, plasmid replicon types, and multilocus sequence types (MLST) was conducted using the Staramr pipeline (v.0.11.0) [22], incorporating ResFinder (database version 13.12.2024), PlasmidFinder (database version 14.11.2024), and MLST (v2.23.0).

The six isolates—GOVDG-1, PLGS-1, PSGS-1, PSCD-1, GORGM-1, and CMCD-1—were derived respectively from green onion, peach leaf, peach orchard soil (two isolates), green onion root, and cow manure (Data file 1) [16]. Their draft genome assemblies consisted of 3 to 7 contigs, with genome sizes ranging from 4.89 to 5.02 Mbp and GC content of approximately 51.9–52.2% (Data file 1) [16]. The total number of predicted coding sequences ranged from 4,541 to 4,761, and all genomes contained 22 rRNA genes and 85–88 tRNA genes. In silico MLST analysis revealed that the isolates belonged to distinct sequence types, including ST4, ST19, ST34, ST198, and ST8316, and they were identified as *S*. *enterica* based on dDDH analysis (Data file 2) [23]. Their serotypes were further predicted and assigned to four serovars: *S*. Typhimurium (GOVDG-1, GORGM-1, and PLGS-1), *S*. I 4 [5],,12:i:- (PSGS-1), *S*. Kentucky (PSCD-1), and *S*. Montevideo (CMCD-1) (Data file 3) [24]. In addition, 3 of them carried acquired AMR genes (Data file 4) [25], most commonly *aph*(3”)-Ib, *aph*(6)-Id, and *tet*(B), as well as at least one plasmid contig (Data file 5) [26]. Virulence genes were detected with the Virulence Factors of Pathogenic Bacteria (Data file 6) [27, 28]. The data were deposited in Figshare and NCBI database (Table 1) [8–13, 16, 23–26, 28].

**Table 1:** Overview of data files/data sets

Label	Name of data file/data set	File types (file extension)	Data repository and identifier (DOI or accession number)
Data file 1	Table S1, Summary of sequencing and annotation results	MS Excel file (.xlsx)	Figshare (10.6084/m9.figshare.28815209) [16]
Data file 2	Table S2, MLST type, and dDDH and ANI values with reference genomes	MS Excel file (.xlsx)	Figshare (10.6084/m9.figshare.28815287) [23]
Data file 3	Table S3, Serotype prediction	MS Excel file (.xlsx)	Figshare (10.6084/m9.figshare.28815548) [24]
Data file 4	Table S4, ResFinder summary	MS Excel file (.xlsx)	Figshare (10.6084/m9.figshare.28829828) [25]
Data file 5	Table S5, PlasmidFinder summary	MS Excel file (.xlsx)	Figshare (10.6084/m9.figshare.28829693) [26]
Data file 6	Table S6, VFDB summary	MS Excel file (.xlsx)	Figshare (10.6084/m9.figshare.28829870) [28]
Data set 1	Raw sequencing data of *S*. *enterica* GOVDG-1, *S*. *enterica* PLGS-1, *S*. *enterica* PSGS-1, *S*. *enterica* PSCD-1, *S*. *enterica* GORGM-1, and *S*. *enterica* CMCD-1	Fastq file (.fastq)	NCBI Sequence Read Archive (SRX28454411 [8], SRX28454412 [9], SRX28454413 [10], SRX28454414 [11], SRX28454415 [12], SRX28454416 [13])
Data set 2	Genome assembly of *S*. *enterica* GOVDG-1	FASTA file (.fasta)/GenBank file (.gbk)	NCBI GenBnk (JBNDEH000000000) [29]
Data set 3	Genome assembly of *S*. *enterica* PLGS-1	FASTA file (.fasta)/GenBank file (.gbk)	NCBI GenBnk (JBNDEI000000000) [30]
Data set 4	Genome assembly of *S*. *enterica* PSGS-1	FASTA file (.fasta)/GenBank file (.gbk)	NCBI GenBnk (JBNDEJ000000000) [31]
Data set 5	Genome assembly of *S*. *enterica* PSCD-1	FASTA file (.fasta)/GenBank file (.gbk)	NCBI GenBnk (JBNDEK000000000) [32]
Data set 6	Genome assembly of *S*. *enterica* GORGM-1	FASTA file (.fasta)/GenBank file (.gbk)	NCBI GenBnk (JBNDEL000000000) [33]
Data set 7	Genome assembly of *S*. *enterica* CMCD-1	FASTA file (.fasta)/GenBank file (.gbk)	NCBI GenBnk (JBNDEM000000000) [34]

## Limitations

The draft nature of the genome assemblies remains fragmented, which may compromise the identification of complete plasmid structures and chromosomal rearrangements. Future studies incorporating hybrid assembly strategies could enhance genomic resolution by enabling accurate reconstruction of plasmid structures and detection of structural variants, thereby improving epidemiological interpretations.

## Data Availability

No datasets were generated or analysed during the current study.

## Abbreviations

AMR: Antimicrobial Resistance
ANI: Average nucleotide identity
dDDH: Digital DNA–DNA Hybridization
NCBI: National Center for Biotechnology Information
ONT: Oxford Nanopore Technologies
VFDB: Virulence factors of pathogenic bacteria
